# *“After those nets are torn, most people use them for other purposes”*: an examination of alternative bed net use in western Kenya

**DOI:** 10.1186/s12936-020-03342-1

**Published:** 2020-07-29

**Authors:** Ellen M. Santos, Jenna E. Coalson, Stephen Munga, Maurice Agawo, Elizabeth T. Jacobs, Yann C. Klimentidis, Mary H. Hayden, Kacey C. Ernst

**Affiliations:** 1 Mel and Enid Zuckerman College of Public Health, University of Arizona, 1295 N Martin Ave, Tucson, AZ 85724 USA; 2 Centre for Global Health Research, Kenya Medical Research Institute, PO Box 1578, Kisumu, 40100 Nyanza Kenya; 3 National Institute for Human Resilience, University of Colorado, University Office Park Building 1867 Suite 200, Boulder, CO 80918 USA

**Keywords:** Malaria, Bed nets, LLIN, Kenya

## Abstract

**Background:**

Alternative long-lasting insecticidal net (LLIN) use for purposes other than sleeping protection from mosquitoes is widely debated as a limitation to successful malaria control efforts, yet rarely rigorously studied.

**Methods:**

A cross-sectional survey of 1217 households in an epidemic highland site and an endemic lowland site in western Kenya collected information on alternative use in three ways: direct observations, participant self-report, and participant reporting of community-level practices. LLIN misuse was defined as use of an intact net for alternative purposes and repurposing as alternatively using an old or damaged net. Associations between households with observed repurposed nets and universal access and household net use were examined.

**Results:**

Households describe repurposing nets when they are torn and/or old. Repurposed nets were observed in 8.1% (52/643) highlands households and 33.0% (184/574) lowlands households. Repurposed nets served as chicken coops (33% highlands, 20% lowlands), fences (37% highlands, 25% lowlands), tree covers (22% lowlands), curtains (3% highlands), covering bathrooms (1.5% highlands, 9% lowlands), and washing sponges (13% lowlands). No association was found between repurposing and universal access or household net use. Misuse was rare. Of 379 repurposed nets, 4 (1.06%) were in good condition with no holes. Of 1,758 active nets, 13 (0.74%) were misused.

**Conclusions:**

Alternative net use in this study involved repurposing rather than misuse. Repurposing was not detrimental to malaria prevention efforts in these communities. Standardized measurement of alternative net use should be used to better understand the practice and its potential impact on the success of malaria interventions.

## Background

Distributing long-lasting insecticidal nets (LLINs) is the primary malaria prevention strategy in sub-Saharan Africa. The use of LLINs (from here, referred to as bed nets or nets) for purposes other than protecting individuals from malaria has been reported in the media [1, 2] and further amplified in the malaria literature [3]. While a point of concern, rigorous investigations of the alternative use of bed nets has not been well explored. It is not clear whether reported misuse can be more accurately described as repurposing that takes place after nets are no longer effective. The Roll Back Malaria (RBM) Social Behavior Change Communication Working Group, the Vector Control Working Group LLIN Priorities Workstream, the Alliance for Malaria Prevention Emerging Issues Working Group, and the President’s Malaria Initiative VectorWorks Project recently put forth standardized definitions for alternative net uses, which encourages accurate, precise, and consistent research and reporting [4], though standardized methods of the best strategies for measurement have yet to be developed.

Repurposing inactive nets (no longer useful for sleeping under) is distinct from misuse [4]. Repurposing can be beneficial (act as some barrier against mosquitoes) or neutral (provide no mosquito barrier). Misuse is the alternative use of an intact net, or the use of a net that causes ecosystem damage from insecticides or overharvesting when used for fishing [4].

Despite public attention, previous research indicates that misuse is uncommon. Eisele and others assert that very little evidence exists to support claims of widespread misuse [5]. In Zambia, the prevalence of misuse (self-reported) of nets for fishing was only 3% among households in an area reliant on fishing [5]. In Sierra Leone, only 5.3% of households self-reported using nets for anything other than protection against mosquitoes [6]. Using predictive modeling, Honjo and others explain that misuse, defined as “alternate net use”, is more likely to occur in contexts of extreme poverty where alternatively using nets is more helpful for the livelihood of the household [7]. Baume and others defined misuse as “using nets for other purposes,” and found it to be uncommon and concentrated in a small minority of communities in Ethiopia [8].

Imprecise definitions of net misuse, alternative purposing, and repurposing dominate the existing literature. Using nets for other purposes has been variably described as “misuse”, “alternative purposing” and “repurposing”. While all three terms indicate use of nets for something other than malaria prevention, they have extremely different implications for malaria control efforts, and self-report likely introduces misclassification due to social-acceptability bias [9]. In fact, many of the “misuse” measurements described in previous research better match definitions of net repurposing [4].

Properly distinguishing between misuse and repurposing is important to understand the drivers and consequences of each phenomenon for malaria prevention. There is evidence suggesting nets are repurposed when households deem them to be old and/or torn [10, 16]. The objective of this work was too better understand the context of net repurposing and misuse in two sites in western Kenya with distinct malaria transmission patterns, and to measure whether net repurposing was predicted by (1) universal access and (2) the percent of household members sleeping under nets.

Existing qualitative information from Ethiopia suggests repurposing is more pervasive than misuse [16], therefore, it was hypothesized that in two regions of differing malaria transmission in western Kenya, alternative net use is more likely a reflection of the repurposing of old nets rather than net misuse. Three measures of alternative net use were reported: direct observations around the home, self-report of alternative uses, and participant reporting of community members. While direct observation of alternative net use is the optimal measurement method, it is difficult to capture sporadic alternative purposes during a single home visit compared to stationary uses such as garden fencing. Additionally, as a community is surveyed over time, households may change their habits or hide alternatively-used nets, leading to underestimates of alternative use. Self-reported alternative use likely underestimates measurements, and reporting what is seen in the community may be biased by what individuals define as community, and probable double-counting of households. While each method has inherent challenges, taken together, they help explain alternative net use in these sites.

## Methods

### Surveys

Cross-sectional surveys were conducted following the rainy seasons in 1217 households among three highlands sublocations (Chepsonoi, Kiborgok, Tindinyo) and 2 lowlands sublocations (Kabar West, Kabar Central) from June to August (Additional file 1) [11]. The highlands sites are situated between 1600–2100 m altitude, experiencing seasonal epidemic malaria while the lowlands sit at 1200 m above sea level and are holoendemic. Details of the parent cross-sectional study involving in-depth household surveys were previously described [11]. Briefly, households were selected from an oversampled random list and were approached for enrollment until a final sample size of 643 highlands households and 574 lowlands households was reached. Detailed descriptions of recruitment, study team training, sample size and power calculations were previously reported [11].

### Definitions

**Fig. 1:**
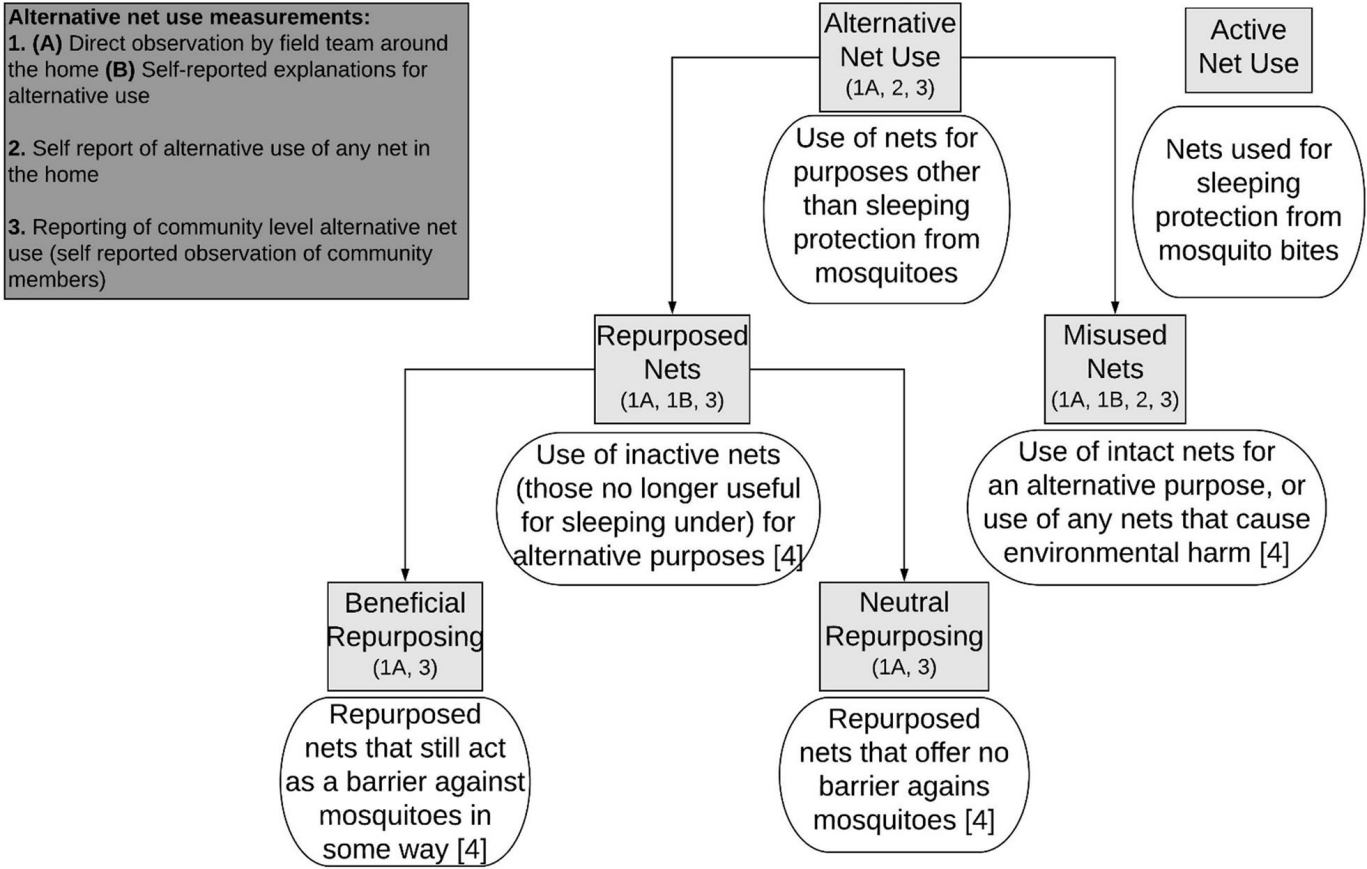
Standardized terminology of the various types of bed net use [8] and how they were measured in this study

Definitions regarding alternative net use are depicted in Fig. 1. Nets were categorized as active or alternatively-used. Direct observation of alternatively used nets (AUNs) was identified through study team observations and inquiry about any AUNs found in or around the household. The study team observed indoor and outdoor areas of each household and recorded all instances of alternative net use. When the study team noted an AUN, they inquired with the participants to understand why the net was being alternatively used. Alternative net use was captured as a binary Yes/No variable with additional qualitative data including the participants’ descriptions of the alternative net use. These qualitative responses were summarized for common responses. Participant self-report was measured through participant open-ended responses detailing purposes of AUNs and self-reported alternative uses of currently active nets. Finally, to measure participant reporting of community-level alternative net use practices, participants who described seeing AUNs in other households around their community was recorded as a binary Yes/No variable.

Households met the WHO-recommended universal access threshold if they had at least one active net for every 2 household members. The percent of household members who slept under a net was calculated using the number who slept under a bed net the previous night divided by the total number of household members.

Household heads were asked about various malaria perceptions including how serious a problem malaria is for their family (Likert scale: not at all serious to extremely serious) and the likelihood of a household member contracting malaria within the next month (Likert scale: not at all likely to extremely likely). Households were categorized into wealth quartiles based on an inverse frequency-weighted asset-ownership index. Household-level ownership of animals, including cattle, sheep, goats, chickens, and dogs, was defined separately. Additionally, households were asked whether they would be able to afford a bed net if one was not provided for free, measured as a binary Yes/No response.

### Statistical analyses

The Pearson Chi-Square test was used to determine whether the distribution of household characteristics and malaria perceptions differed between households with and without AUNs. To assess associations between the presence of AUNs and universal access and members’ net use, univariate logistic regression models were used. Logistic regression assumptions were tested where appropriate, including independent observations and linearity in the log-odds. Because a household’s local community likely influences both the selected independent variables and alternative use, analyses were adjusted for sublocation. Stata v12 was used (College Station, TX) and α = 0.05 for all analyses.

## Results

### Demographics

Population distributions of key study variables are reported in Table 1. Highlands households tended to be larger than lowlands households. There were 202 (31.6%) highlands households owning no nets compared to 11 (1.9%) in the lowlands. Most households reported no problems using a net. Households in both sites were relatively evenly distributed among wealth quartiles, though 198 (30.9%) highlands households reported inability to afford a new net compared to 73 (12.9%) lowlands households. There were 402 (62.7%) highlands households and 160 (27.9%) lowlands households that did not meet universal access criteria. However, between 76–100% of household members used a net among 515 (92.3%) households in the lowlands compared to 293 (46.3%) households in the highlands.

**Table 1:** Household characteristics and malaria perceptions comparing households with and without observed repurposed bed nets

		Highlands				Lowlands			
	Total	Any repurposing (n = 52)	No repurposing (n = 587)	*P* value^a^	Total	Any repurposing (n = 184)	No repurposing (n = 374)	*P* value^a^	
People per household	(n = 639)			0.629	(n = 558)			0.183	
1–3	240 (37.6)	21 (8.8)	219 (91.3)		372 (66.7)	120 (32.3)	252 (67.7)		
4–6	293 (45.9)	25 (8.5)	268 (91.5)		164 (29.4)	53 (32.3)	111 (67.7)		
≥ 7	106 (16.6)	6 (5.7)	100 (94.3)		22 (3.9)	11 (50.0)	11 (50.0)		
Nets per household	(n = 639)			0.236	(n = 558)			0.470	
0	202 (31.6)	11 (5.4)	191 (94.6)		11 (1.9)	2 (20.0)	8 (80.0)		
1	167 (26.1)	17 (10.2)	150 (89.8)		309 (55.4)	95 (30.7)	214 (69.3)		
2	152 (23.8)	15 (9.9)	137 (90.1)		179 (32.1)	65 (36.3)	114 (63.7)		
≥ 3	118 (18.5)	7 (8.2)	78 (91.8)		60 (10.8)	16 (35.6)	29 (64.4)		
How serious a problem malaria is for the family	(n = 637)			0.383	(n = 557)		0.938		0.273
Not at all serious	22 (3.5)	4 (18.2)	18 (81.8)		5 (0.9)	2 (40.0)	3 (60.0)		
Slightly serious	357 (56.0)	26 (7.3)	331 (92.7)		143 (25.7)	43 (30.1)	100 (69.9)		
Somewhat serious	189 (29.7)	16 (8.5)	173 (91.5)		100 (18.0)	33 (33.0)	67 (67.0)		
Serious	64 (10.0)	5 (7.8)	59 (92.2)		283 (50.8)	96 (33.9)	187 (66.1)		
Extremely serious	5 (0.8)	1 (20.0)	4 (80.0)		26 (4.7)	9 (34.6)	17 (65.4)		
Problems ever using a net	(n = 534)			0.184	(n = 538)		0.696		0.438
Yes	43 (8.1)	6 (14.0)	37 (86.0)		72 (13.4)	25 (34.7)	47 (65.3)		
No	491 (91.9)	40 (8.1)	451 (91.9)		466 (86.6)	151 (32.4)	315 (67.6)		
Wealth quartile	(n = 628)			0.809	(n = 564)				0.234
1st Quartile	160 (25.5)	12 (7.5)	148 (92.5)		118 (20.9)	34 (28.8)	84 (71.2)		
2nd Quartile	211 (33.6)	18 (8.5)	193 (91.5)		103 (18.3)	26 (25.2)	77 (74.8)		
3rd Quartile	128 (20.4)	8 (6.3)	120 (93.8)		156 (27.7)	55 (35.3)	101 (64.7)		
4th Quartile	129 (20.5)	12 (9.3)	117 (90.7)		187 (33.2)	69 (36.9)	118 (63.1)		
Ability to afford a net	(n = 640)			0.753	(n = 567)				0.888
Yes	442 (69.1)	36 (8.1)	403 (91.2)		494 (87.1)	157 (31.8)	322 (65.2)		
No	198 (30.9)	15 (7.6)	182 (91.9)		73 (12.9)	23 (31.5)	49 (67.1)		
Sublocation	(n = 642)			0.038*	(n = 574)				0.284
Chepsonoi	221 (34.4)	22 (10.0)	199 (90.0)						
Kiborgok	204 (31.8)	12 (5.9)	192 (94.1)						
Tindinyo	217 (33.8)	18 (8.3)	199 (91.7)						
Kabar Central					316 (55.1)	106 (33.5)	210 (66.5)		
Kabar West					258 (44.9)	78 (30.2)	180 (69.8)		
Universal access	(n = 641)			0.211	(n = 574)				0.668
Yes	239 (37.3)	21 (8.8)	218 (91.6)		414 (72.1)	134 (32.4)	272 (65.7)		
No	402 (62.7)	31 (7.7)	369 (91.8)		160 (27.9)	50 (31.3)	102 (63.8)		
% HH slept under a net	(n = 633)			0.115	(n = 558)				0.610
0–25%	227 (35.9)	11 (4.8)	216 (95.2)		15 (2.7)	4 (26.7)	11 (73.3)		
26–50%	67 (10.6)	8 (11.9)	59 (88.1)		12 (2.2)	2 (16.7)	10 (83.3)		
51–75%	46 (7.3)	5 (10.9)	41 (89.1)		16 (2.9)	5 (31.2)	11 (68.8)		
76–100%	293 (46.3)	28 (9.6)	265 (90.4)		515 (92.3)	173 (33.6)	342 (66.4)		
Own cattle	(n = 639)			0.974	(n = 558)				0.852
Yes	355 (55.6)	29 (8.2)	326 (91.8)		288 (51.6)	96 (33.3)	192 (66.7)		
No	284 (44.4)	23 (8.1)	261 (91.9)		270 (48.4)	88 (32.6)	182 (67.4)		
Own sheep	(n = 639)			0.992	(n = 558)				0.754
Yes	74 (11.6)	6 (8.1)	68 (91.9)		117 (21.0)	40 (34.2)	77 (65.8)		
No	565 (88.4)	46 (8.1)	519 (91.9)		441 (79.0)	144 (32.7)	297 (67.3)		
Own goats	(n = 639)			0.191	(n = 558)				0.028*
Yes	56 (8.8)	2 (3.6)	54 (96.4)		175 (31.4)	69 (39.4)	106 (60.6)		
No	583 (91.2)	50 (8.6)	533 (91.4)		383 (68.6)	115 (30.0)	268 (70.0)		
Own chickens	(n = 638)			0.117	(n = 558)				0.000*
Yes	497 (77.9)	45 (9.1)	452 (90.9)		441 (79.0)	163 (37.0)	278 (63.0)		
No	141 (22.1)	7 (5.0)	134 (95.0)		117 (21.0)	21 (17.9)	96 (82.1)		
Own dogs	(n = 639)			0.004*	(n = 558)				0.212
Yes	155 (24.3)	4 (2.6)	151 (97.4)		325 (58.2)	114 (35.1)	211 (64.9)		
No	484 (75.7)	48 (9.9)	436 (90.1)		233 (41.8)	70 (30.0)	163 (70.0)		
HH reports seeing others repurpose bed nets	(n = 637)			0.000*	(n = 557)				0.011*
Yes	450 (70.6)	48 (10.7)	402 (89.3)		525 (94.3)	180 (34.3)	345 (65.7)		
No	187 (29.4)	4 (2.1)	183 (97.9)		32 (5.7)	4 (12.5)	28 (87.5)		

^a^Pearson’s Chi-Square test

*Statistically significant *p* < 0.05

### Observation of alternatively-used bed nets

Alternative net use was less common in the highlands than the lowlands. Of 643 highlands households, there were 52 (8.1%) observed with AUNs, while the lowlands proportion was higher at 184 (33.0%) of 574 households. AUNs clustered in households, as there were 67 AUNs among 52 highlands households and 312 AUNs among 184 lowlands households. AUNs also clustered at the sublocation level. Of the three highlands sublocations, Chepsonoi had a significantly higher proportion compared to Kiborgok (10.0 vs*.* 5.9% households, *p* = 0.038). The proportion was similar for the two lowlands sublocations, Kabar Central and Kabar West (Table 1). There were only 11 households (1.7%) in the highlands and 3 households (0.5%) in the lowlands that had an AUN, and in which no household members slept under a net.

**Fig. 2:**
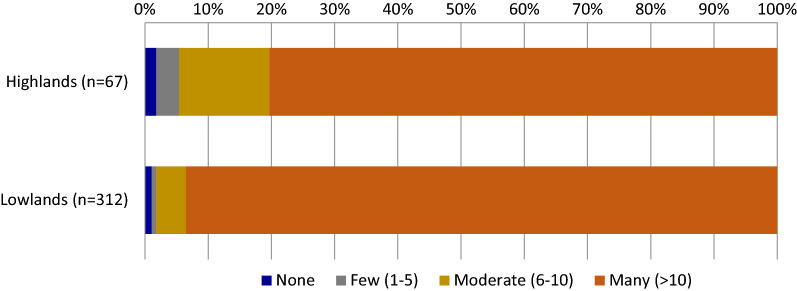
Holes observed among repurposed bed nets

**Fig. 3:**
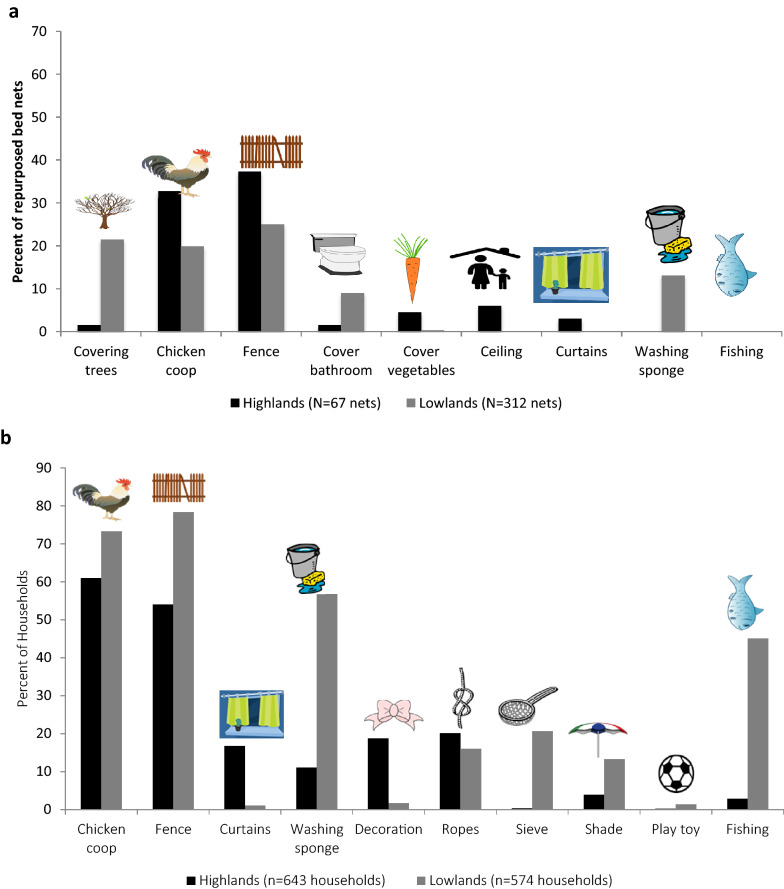
Uses of AUNs as observed by the study team (**a**) and by study participants (**b**). All images are royalty-free images from publicdomainvectors.org

In both sites, most alternatively-used nets were visibly damaged, with 10 or more holes in at least 80% of the observed nets (Fig. 2). Most AUNs in the highlands were used outdoors (77.6%) (Fig. 3a). The most common functions of observed AUNs were as chicken coops or fences (33 and 37%, respectively). AUNs indoors were used as curtains or to cover ceilings or bathrooms. Similarly in the lowlands, most AUNs (69.8%) were used outdoors, covering trees (22%), as chicken coops (20%), or as fences (25%) (Fig. 3a). Indoor AUNs covered bathrooms or were cut up and used as washing sponges.

Twenty-six highlands households (50%) and 127 (69%) lowlands households with AUNs reported why they alternatively used nets. Most reported they repurposed nets because they were no longer serviceable as sleep protection (Table 2). Of these repurposed nets in the highlands, 7 (10.4%) were beneficially repurposed and 60 (89.6%) were neutrally repurposed. In the lowlands, 28 (9.0%) were repurposed beneficially and 284 nets (91.0%) were neutral.

**Table 2:** Household-reported reasons for repurposing bed nets

	Highlands n (%) N = 26 households	Lowlands n (%) N = 127 households	Representative quote
Net was torn/too many holes	10 (38.5)	23 (18.1)	*“The [net] had many holes that could not be sewn up”*
Net was old and worn out	5 (19.2)	78 (61.4)	*“[The net] had lasted a long period of time…more than 5 years therefore [we] decided to use it as a garden fence”*
Net was old and had holes	7 (26.9)	14 (11.0)	*“[The net] had many holes and [was] old”*
Damage by outside factors	1 (3.8)	3 (2.4)	*“Bed net was eaten by a rat”*
Needed to protect crops	2 (7.7)	3 (2.4)	*“[The net was used] to prevent hens from attacking the vegetables”*
Did not want to throw away	1 (3.8)	2 (1.6)	*“[It is] better that way instead of throwing away after it was torn”*
Other	0	4 (3.1)	*“After those nets are torn most people use them for other purposes”*

### Participant self-report

Though there were insufficient data to determine insecticidal efficacy, only 1 of 67 repurposed nets in the highlands (1.8%) and 3 of 312 repurposed nets in the lowlands (1.1%) were visibly intact, suggesting misuse is uncommon (Fig. 2). The households that owned these misused nets did not provide a response to explain why they were not using them for malaria protection. Bed net misuse may also be sporadic. Among 874 actively used nets inside homes in the highlands, household members only reported that 7 (0.8%) had ever been used for something other than mosquito protection while sleeping. Two households reported that these currently active nets had been used previously as chicken coops, and four households reported the nets had been used as fencing around vegetables. Similarly, previous alternative uses were reported for 6 (0.7%) of 884 functional nets in the lowlands, though the purposes were not specified.

### Reports of alternative net use in the community

Participant reporting on AUNs in their community corresponded well with trends reported in observations by the study team in both sites, all sublocations, and across net uses. There were 525 (94.3%) lowlands households that reported observing others with AUNs in their community compared to 450 (70.6%) highlands households (Table 1). Participants substantiated the observed differences between sublocations with more households in Chepsonoi reporting community members with AUNs (86.0%) than in Kiborgok (49.0%). Slightly more households reported observing AUNs in their community in Kabar Central (97.8%) compared to Kabar West (88.8%). Participants observed similar uses for nets as the study team; chicken coops, fences, and washing sponges among the most common. Additional uses were reported, including fishing, decoration, ropes, and sieves (Fig. 3b).

### Characteristics of households with observed net repurposing

There were no statistically significant differences in the distributions of any household characteristics between households with and without repurposed nets in both sites (Table 1). Most households reported malaria is a slightly or somewhat serious problem for their family (highlands), and a slightly-to-serious problem (lowlands). Animal ownership varied slightly between the highlands and lowlands (Table 1). Chickens were the most commonly owned animal in both sites.

### Factors associated with observed repurposing

**Fig. 4:**
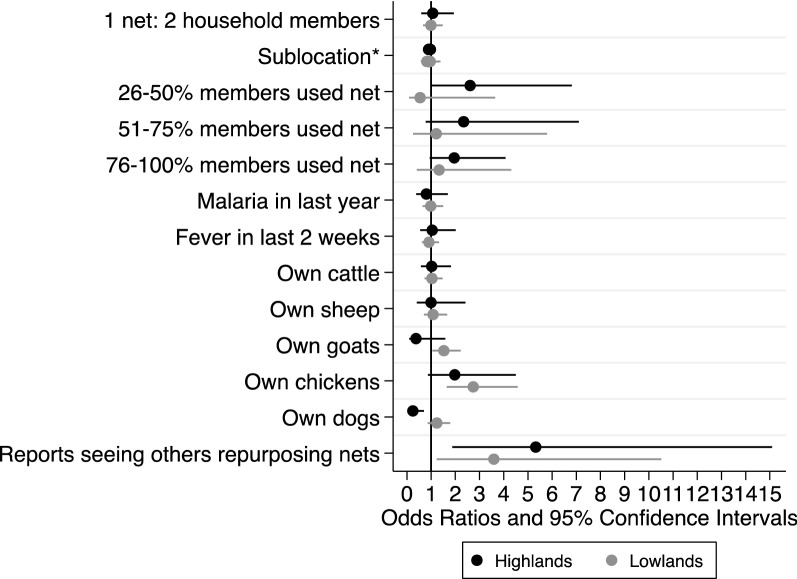
Univariate logistic regression results (odds ratios and 95% confidence intervals) assessing factors associated with the presence of bed net repurposing at the household level, adjusted for sublocation

Households with AUNs were not significantly more likely to meet universal access than those without AUNs in either the highlands (40.4 vs. 37.1%, *p* = 0.211) or the lowlands (33 vs. 32.9%, *p* = 0.668). There were 27 highlands households (4.2%) and 13 lowlands households (2.3%) that repurposed nets despite having at least one household member who did not use a net. In both sites, there was no association between observed net repurposing and universal access (OR highlands: 1.0, lowlands: 0.99) or members’ net use (Fig. 4). Though not statistically significant, repurposing was inversely related with proportion of household members using a net in the highlands. This trend was not observed in the lowlands.

Animal ownership was positively associated with repurposing in the lowlands, where households with repurposed nets had 1.53 times the odds of owning goats and 2.74 times the odds of owning chickens compared to households with no repurposed nets. Notably, households that repurposed nets were significantly more likely to report observing other community households repurposing nets [OR: 7.27 (2.22, 23.79) highlands; OR: 3.59 (1.23, 10.51) lowlands] compared to households with no repurposed nets (Fig. 4).

## Discussion

### Observation of repurposed bed nets

There were 8.1 and 33.0% of households with observable repurposed nets in the highlands and lowlands, respectively. Repurposing nets is reportedly common throughout Kenya [12]. Nets were repurposed for multiple uses indoors and outdoors, particularly for fencing, chicken coops, protecting crops, covering bathrooms, serving as curtains, or cut up as cleaning rags. Of repurposed nets, 89.6 and 91.0% in the highlands and lowlands, respectively, are classified as neutral as their new purpose does not protect household members from mosquitoes but also does not cause damage or harm [4]. Many neutral nets were repurposed to help store, dry, or otherwise secure food. Repurposing for food security purposes has been demonstrated in other studies [13, 14]. Few nets were beneficially repurposed (still offered protection against mosquitoes). Households that use bed nets for sleeping while also beneficially repurposing old nets as screens, curtains, or ceiling covers [8, 12, 15] may have better mosquito protection than households that dispose old nets or neutrally repurpose them. Kibe and others suggest development of repurposing and disposal guidelines to promote beneficial repurposing [12].

### Reported reasons for bed net repurposing

Nearly all households with AUNs reported the nets were too old and/or damaged to be used for sleeping under. Qualitative studies in Ethiopia and Kenya further support this finding [12, 16]. The netting is considered strong and durable for other uses [16]. While repurposing almost always occurs when nets are perceived as no longer useful for sleeping protection, households’ definitions of usability may vary [10, 16]. Some households may consider nets with only a few holes to be too damaged [15, 17]. Net age at repurposing can be greater than two years [18]. A longitudinal study following LLINs post-distribution in Zambia found the mean net age of discarded or repurposed nets was 18 months [19]. Though it is unknown whether holes appeared in repurposed nets before or after they were repurposed, nearly all repurposed nets in both the highlands and lowlands had many holes or were fragments of nets. Efforts promoting net repair may foster longer use for mosquito protection before they are discarded or repurposed. However, it appears that the benefits of alternative use are not outweighed by the benefits of mosquito protection, at least until nets are worn out and ineffective.

### Reports of alternative net use in the community

Another indicator of alternative net use was participant perception of frequency in their community. Viewing alternative use as socially acceptable or normalized may influence whether households repurpose nets, as households were significantly more likely to have repurposed nets if they observed other households in the community alternatively using nets. Repurposing is also often concentrated in communities [8]. In Senegal, study participants reported seeing bed nets used for alternative purposes in some communities, but not others [15], emphasizing the clustered nature of alternative net use. Social acceptability is associated with other aspects of net use, particularly the desire to keep nets from appearing dirty [20, 21]. Rather than throwing old, worn nets away, households observe others finding innovative uses for them, see that the practice is acceptable, and find ways to make their nets useful.

### Bed net misuse

Net misuse was also measured. Using an intact, functional net for a purpose other than mosquito protection while sleeping was rare. Of repurposed nets, one in the highlands and three in the lowlands had no holes, though these households did not report reasons for alternatively using these nets. Additionally, among nets within the home, less than 1% of functioning bed nets were reported as having been alternatively used in both sites. Previous reports also found rare misuse [5] and suggest it occurs for economically beneficial reasons [6, 22], particularly where misused for fishing [13, 14, 23].

There were no observations or self-reported instances of misused nets for fishing, though interestingly nearly 50% of households in the lowlands reported seeing others using nets for fishing. It is unclear whether misuse for fishing is actually occurring frequently in these sites, or whether participants were referring to unsampled geographic locations when they noted that they have seen others use nets for fishing. It is likely the latter explanation, as these study sites are not situated near large bodies of water and fishing is not a major economic activity. Most areas where bed nets are misused for fishing are heavy fishing communities [14, 18, 23–25] near large bodies of water including coastal areas, the African Great Lakes, and large rivers [26]. These study sites, like others where non-fishing activities drive the economy, report rare misuse of this kind [5, 6, 8]. Due to environmental harm caused by fishing with ITNs or LLINs [27–30], misusing nets for fishing is a serious problem that should be addressed [4]. While misusing nets for fishing is an environmental problem, it is not clear if misuse is a problem for malaria prevention efforts including net ownership and use. It is not always clear whether households using nets for fishing also sleep under intact nets [14].

### Impact of net repurposing on malaria prevention efforts

Though common, bed net repurposing only poses a problem to malaria prevention efforts if households that repurpose nets do not meet universal access, if household members do not use nets, or if the repurposing leads to community-level shortages of nets. There was no significant association between household net repurposing and household universal access or members’ net use. While there were more households with repurposed nets in the lowlands than in the highlands, more household members also slept under nets in the lowlands, supporting reports that repurposing is unlikely detrimental for household net use for mosquito prevention [5]. In both sites, few households with repurposed nets had household members who did not sleep under a net. Though sleeping under a torn, old net may still offer some protection against mosquitos if there is residual insecticide [31], most households with repurposed nets also used active nets for sleeping. In a study where misuse for fishing was very common, more than 80% of individuals using nets for fishing reported also using nets in the household [14]. As more net distributions occur, households are likely to attain newer nets to replace worn nets that are then repurposed in economically beneficial ways to the household [12]. In contrast to the lowlands, there was a non-significant, though interesting trend suggesting households with fewer individuals sleeping under nets were more likely to have repurposed nets in the highlands than households where nearly everyone used a net. Even in lowlands households not owning enough nets, several household members crowded under a single net, while this occurred infrequently in the highlands [11]. This suggests potential differences in net use and repurposing behaviours between areas of year-round and seasonal *Plasmodium* transmission.

## Limitations

Direct measurement of net misuse was difficult. Misuse was defined as repurposed nets that were in physically good condition with no holes, or as nets used as intended in the household but reported to have ever been used for an alternative purpose. While the study team was able to directly observe repurposed nets, the age or condition of the net when first repurposed is unknown. It is possible that nets were first repurposed when they were still viable for intended use. Insecticidal efficacy of the nets was not measured, leaving no ability to comment on net efficacy other than through physical net condition. However, nearly all participants discussed repurposing nets once they were old or damaged, though not all households with observed AUNs provided a response as to *why* they repurposed nets.

Additionally, the occurrence of alternative net use may have been underestimated. As word spreads through the community regarding a survey of bed nets, some households may begin to change their habits or hide their nets if they feel they should not be using them for alternative purposes. However, this underestimation is likely minimal, as no evidence of reduction in the proportion of observed alternative use over the study period in each community was found.

## Conclusions and recommendations

Bed net misuse was very rare in these study sites, while net repurposing was more common. Bed nets were repurposed for various outdoor and indoor purposes such as fencing for chicken coops, or cut up into washing sponges. The presence of repurposing was not associated with universal access or household members’ net use. Thus, net repurposing does not appear to be detrimental for malaria prevention efforts. Future assessments of bed net misuse and repurposing should describe each occurrence according to the Roll Back Malaria Consensus Statement [8] to ensure the same definitions are used for comparability between studies and regions. The authors recommend development of standardized methods for measuring alternative net use to increase comparability across studies. The authors propose the following methods and variables for data collection:

1. Direct observation of alternative net use of observer and household-reported data including:
  - Description of alternative purpose of the net
  - Description of net condition at time of observation
  - Explanation for alternative use of net
  - Description of net condition at time of first alternative use
  - Time net was acquired, and time net was first alternatively-used
2. Inquire about additional nets not visible during the observation visit, and determine their alternative-use status and purpose.
3. Improve self-report of misuse of active (functional) nets. In the present study, participants responded whether active nets were ever used for alternative purposes. For more informative qualitative data, the authors recommend re-phrasing similar survey items to, “If this net is ever used for another purpose, what would you use it for?”

## Supplementary information

**Additional file 1.** Map depicting the location of the three highlands study sites (Chepsonoi, Kiborgok, Tindinyo) and the two lowlands study sites (Kabar West and Kabar Central).

## Data Availability

The datasets used and/or analysed during the current study are available from the corresponding author on reasonable request.
