# Supplementary material for: “After those nets are torn, most people use them for other purposes”: an examination of alternative bed net use in western Kenya
Source: Malar J. 2020 Jul 29;19:272. doi: 10.1186/s12936-020-03342-1 (PMC7390200; doi:10.1186/s12936-020-03342-1)
Supplement: Supplementary file 1 — Additional file 1. Map depicting the location of the three highlands study sites (Chepsonoi, Kiborgok, Tindinyo) and the two lowlands study sites (Kabar West and Kabar Central). [file 12936_2020_3342_MOESM1_ESM.docx]

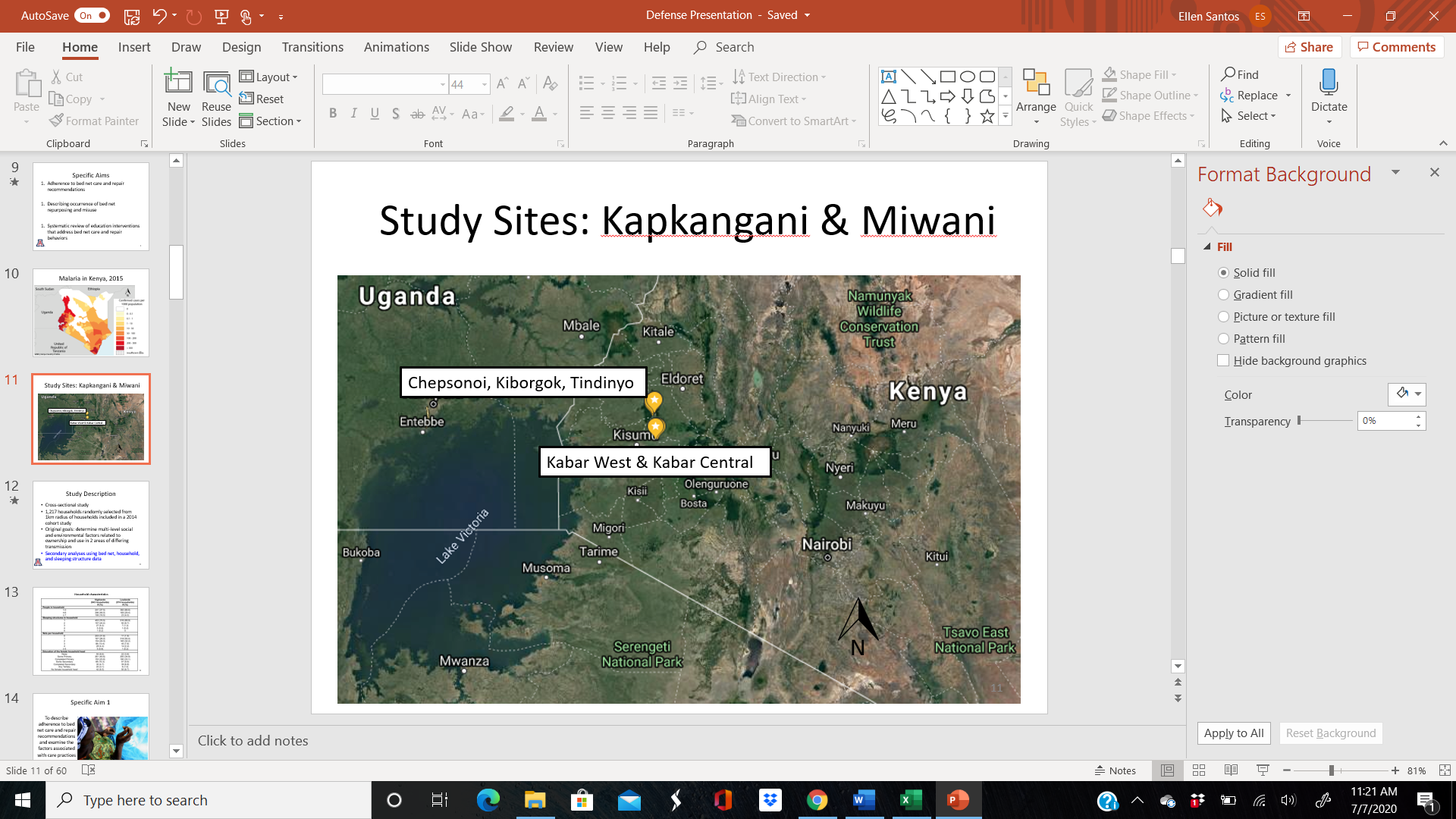


Additional File 1. Map depicting the location of the three highlands study sites (Chepsonoi, Kiborgok, Tindinyo) and the two lowlands study sites (Kabar West and Kabar Central).
